# Establishment of Collagen: Hydroxyapatite/BMP-2 Mimetic Peptide Composites

**DOI:** 10.3390/ma13051203

**Published:** 2020-03-07

**Authors:** Liane Schuster, Nina Ardjomandi, Marita Munz, Felix Umrath, Christian Klein, Frank Rupp, Siegmar Reinert, Dorothea Alexander

**Affiliations:** 1 Department of Oral and Maxillofacial Surgery, University Hospital Tübingen, 72076 Tübingen, Germany; liane.schuster84@gmail.com (L.S.); n.ardjomandi@googlemail.com (N.A.); marita.munz@med.uni-tuebingen.de (M.M.); felix.umrath@med.uni-tuebingen.de (F.U.); Siegmar.reinert@med.uni-tuebingen.de (S.R.); 2 Dental Practice Meller Zahngesundheit, 71332 Waiblingen, Germany; 3 Department of Conservative Dentistry, Periodontology and Endodontology, University Hospital Tübingen, 72076 Tübingen, Germany; 4 Section Medical Materials Science & Technology, University Hospital Tübingen, 72076 Tübingen, Germany; Frank.Rupp@med.uni-tuebingen.de

**Keywords:** collagen/hydroxyapatite scaffolds, BMP-2 mimicry peptides, electrostatic binding on hydroxyapatite, quartz crystal microbalance

## Abstract

Extensive efforts were undertaken to develop suitable biomaterials for tissue engineering (TE) applications. To facilitate clinical approval processes and ensure the success of TE applications, bioinspired concepts are currently focused on. Working on bone tissue engineering, we describe in the present study a method for biofunctionalization of collagen/hydroxyapatite composites with BMP-2 mimetic peptides. This approach is expected to be fundamentally transferable to other tissue engineering fields. A modified BMP-2 mimetic peptide containing a negatively charged poly-glutamic acid residue (E7 BMP-2 peptide) was used to bind positively charged hydroxyapatite (HA) particles by electrostatic attraction. Binding efficiency was biochemically detected to be on average 85% compared to 30% of BMP-2 peptide without E7 residue. By quartz crystal microbalance (QCM) analysis, we could demonstrate the time-dependent dissociation of the BMP-2 mimetic peptides and the stable binding of the E7 BMP-2 peptides on HA-coated quartz crystals. As shown by immunofluorescence staining, alkaline phosphatase expression is similar to that detected in jaw periosteal cells (JPCs) stimulated with the whole BMP-2 protein. Mineralization potential of JPCs in the presence of BMP-2 mimetic peptides was also shown to be at least similar or significantly higher when low peptide concentrations were used, as compared to JPCs cultured in the presence of recombinant BMP-2 controls. In the following, collagen/hydroxyapatite composite materials were prepared. By proliferation analysis, we detected a decrease in cell viability with increasing HA ratios. Therefore, we chose a collagen/hydroxyapatite ratio of 1:2, similar to the natural composition of bone. The following inclusion of E7 BMP-2 peptides within the composite material resulted in significantly elevated long-term JPC proliferation under osteogenic conditions. We conclude that our advanced approach for fast and cost-effective scaffold preparation and biofunctionalization is suitable for improved and prolonged JPC proliferation. Further studies should prove the functionality of composite scaffolds in vivo.

## 1. Introduction

Initial attempts to develop mesenchymal stromal stem cells (MSCs)-based therapies were met with limited success. The injected cells showed poor survival and/or integration into host tissues [1]. Future cell delivery systems should take inspiration from the specialized in vivo microenvironments of cell niches, which activate stem cell populations.

In bone tissue engineering, calcium phosphate (CaP) biomaterials are frequently chosen as scaffolds for the delivery of mesenchymal stromal cells (MSCs) to induce new bone formation at the defect site. Based on this fact, the dissolution of calcium and phosphate ions from the solid phase of CaP biomaterials into the surrounding environment of used cells should be taken into consideration.

In vitro studies have shown that Ca^2+^ ions positively influence proliferation and morphology of human periosteal-derived stem cells (hPDCs) [2] or the osteogenesis of osteoblasts [3,4,5].

Hydroxyapatite (HA) is chemically similar to the inorganic component of the natural bone and exhibits excellent biocompatibility with soft tissues. These features make it to an ideal candidate for dental and orthopedic implants [6]. Besides of hydroxyapatite as the main inorganic component of natural bone, type-I collagen represents the main organic component which initiates and orientates the growth of carbonated apatite mineral [7]. In order to achieve a natural scaffold, we were inspired by the natural composition of bone and combined both components. In a recent comparative study, nano-hydroxyapatite/collagen composites showed higher Young’s moduli and a higher induction of late osteogenesis marker expression compared to natural bone ceramic/collagen scaffolds [8]. The combination of a human-like collagen with nano-hydroxyapatite seems to elicit excellent mechanical and biological properties [9].

In previous works, we treated the surface of β-tricalcium phosphate scaffolds in order to be able to biofunctionalize it and to mimic a more natural microenvironment for the colonializing periosteal cells [10,11]. However, solvents and crosslinkers are required for its immobilization due to missing functional groups on the surface of β-tricalcium phosphate. In contrast, the surface of hydroxyapatite is much more reactive and simple electrostatic binding of proteins could be a tempting solution for immobilization [12]. Bone morphogenetic protein-2 (BMP-2) is a known potent inducer of osteogenesis. Since growth factors are very expensive because of their laborious production, and studies have questioned the cost-effectiveness of current BMP-2 treatments [13], appropriate delivery of growth factors is needed to be investigated meticulously. Considering future clinical applications and in order to facilitate regulatory affairs, tissue engineering constructs should gain increasing simplicity. Furthermore, the use of short peptides instead of recombinant proteins shall be pursued to avoid adverse effects [14]. For instance, Lee and co-workers could modulate the retention of osteogenic peptides derived from BMP-2 or BMP-7 by polydopamine-mediated immobilization of electrospun nanofibers. This approach for the loading of activating factors is very simple and the need of relatively low amounts to induce bone regeneration might be the result [15,16].

New strategies involve the application of peptides containing binding sites for respective receptors instead of proteins. A synthetic peptide corresponding to the residues 73–92 of the knuckle epitope of BMP-2 has been shown to stimulate bone precursor cells to induce calcification [17]. Furthermore, the use of this mimetic peptide encapsulated in thermosensitive hydrogels for minimally invasive surgery of bone repair was taken into consideration [18,19].

In the present work, we modified the mimetic BMP-2 peptide by adding a poly-glutamic acid residue (E7 Tag), this conferring accumulated negative charges and facilitating binding capacities of peptides to positively charged surfaces such as hydroxyapatite. Furthermore, this method for immobilization needs no chemical reactions or organic solvents, which can influence biomolecule activity and/or physicochemical integrity of the biomaterials and scaffolds [20]. This paper describes the development of collagen/HA composites biofunctionalized with E7 BMP-2 mimicry peptides and their effect on encapsulated jaw periosteal cells (JPCs).

## 2. Materials and Methods

### 2.1. Cell Isolation and Culture of JPCs

JPCs derived from three donors were included in this study in accordance with the local ethical committee (approval number 194/2008BO2) and after obtaining written informed consent. The jaw periosteal tissue was cut in small pieces with a scalpel and enzymatically digested with type XI collagenase (1500 U/mL, Sigma-Aldrich, Steinheim, Germany) for 90 min. Enzymatically isolated cells were expanded in DMEM/F12 (Dulbecco’s Modified Eagle Medium) + 10% fetal calf serum (FCS) for up to 4–5 passages until used in passage 5–6 for all proliferation and differentiation assays. JPCs were therefore cultured within different collagen/HA composites as described in the following sections. For 96-well plates, 5 × 10^4^ cells were mixed with 100 µL collagen/HA gel. For differentiation experiments, culture medium was supplemented with 10 mM β-glycerophosphate, 100 mM L-ascorbic acid 2-phosphate, and 4 mM dexamethasone (Sigma-Aldrich, Darmstadt, Germany) for the indicated time points.

### 2.2. Preparation of Collagen/Hydroxyapatite (HA) Composites

**Figure 1:**
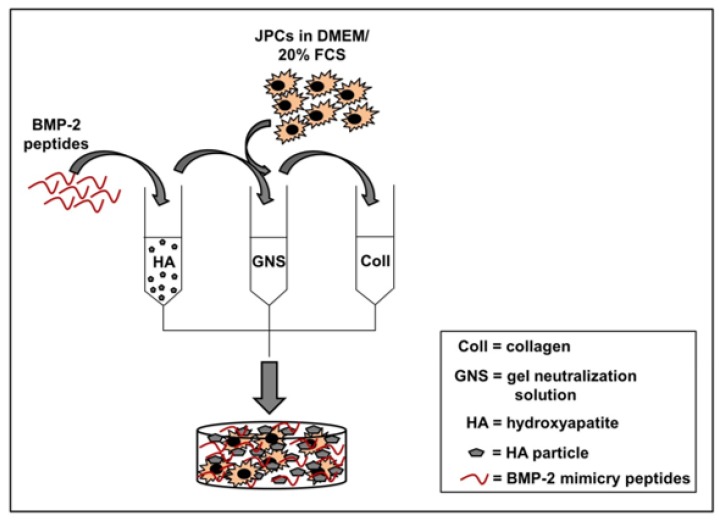
Preparation of collagen/HA composites. For the preparation of 3D composite scaffolds, BMP-2 mimicry peptides (200 µg peptide per 10 mg HA) were added to hydroxyapatite (HA) and incubated for 2 h for binding. The functionalized HA was mixed with a gel neutralizing solution (GNS) and jaw periosteal cells (JPCs) in DMEM/20% FCS were added. This mixture was combined with rat type I collagen (rat type I collagen, provided by Amedrix GmbH, Esslingen, Germany) solution. Direct distribution of the obtained solution in cell culture plates followed.

For the preparation of collagen/HA composites, the required amount of HA (nanoXIM HAp 203 powder as micrometric aggregates of hydroxyapatite nanoparticles (particle size 10 µm, Fluidinova, Moreira da Maia, Portugal) was weighted and after one wash step in TBS (tris buffered saline), an overnight incubation in TBS followed for equilibration. Thereafter, the TBS/HA solution was centrifuged for 10 min at 5000 rpm. TBS supernatant was discarded and a peptide solution (in TBS, 200 µg peptide per 10 mg HA) was added and incubated with HA for 2 h. The respective volume of the collagen/gel neutralization solution (GNS) (as listed in Table 1) was supplied (draft of composite preparation, see Figure 1). BMP-2 mimicry peptides corresponding to residues 73–92 of BMP-2 with or without E7-Tag were provided from the company Biomatik (Cambridge, Canada).

JPCs were detached from the culture plates by trypsin, cells were counted and cell concentration was adjusted in DMEM/20% FCS for the pipetting of 5 × 10^4^ per 100 µL collagen gel (Amedrix GmbH, Esslingen, Germany) per well of the 96-well plates. The GNS/HA solution was then mixed with the cell suspension (in DMEM/20% FCS) and added to the collagen solution and mixed with a syringe. 100 µL were then pipetted into the wells of a 96 well plate using a multichannel pipette. During the next 30 min, gels were allowed to polymerize and 200 µL of culture medium was added. The day after, medium change followed and normal (Co) and osteogenic media (Ob) were pipetted to the wells.

### 2.3. Biochemical Quantification of BMP-2 Peptide Binding to HA

**Figure 2:**
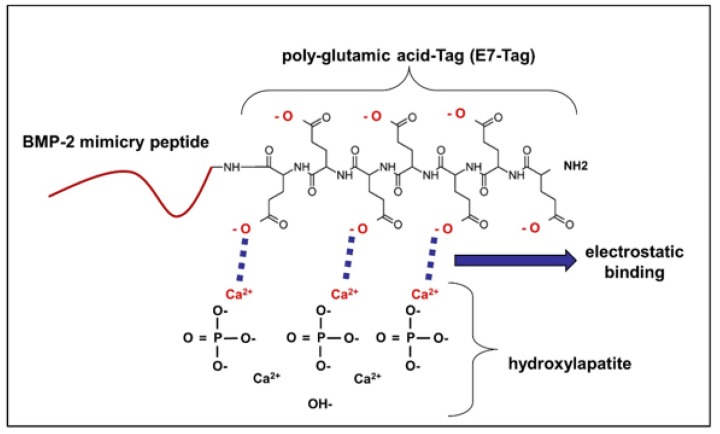
Principle of electrostatic binding of BMP-2 mimicry peptides to HA. BMP-2 mimicry peptides bind through their negative charges to the positively charged Ca ions of hydroxyapatite (HA). E7 BMP-2 peptides contain additionally a poly-glutamic acid residue conferring them the possibility to strongly bind electrostatically to HA on this site.

As illustrated in Figure 2, BMP-2 mimicry peptides bind through negative charges of the glutamic acid residues (E7 Tag) to the positively charged Ca^2+^ ions of the hydroxyapatite (HA) by electrostatic interactions. To prove this chemical binding, two approaches were performed as described in the following.

**Figure 3:**
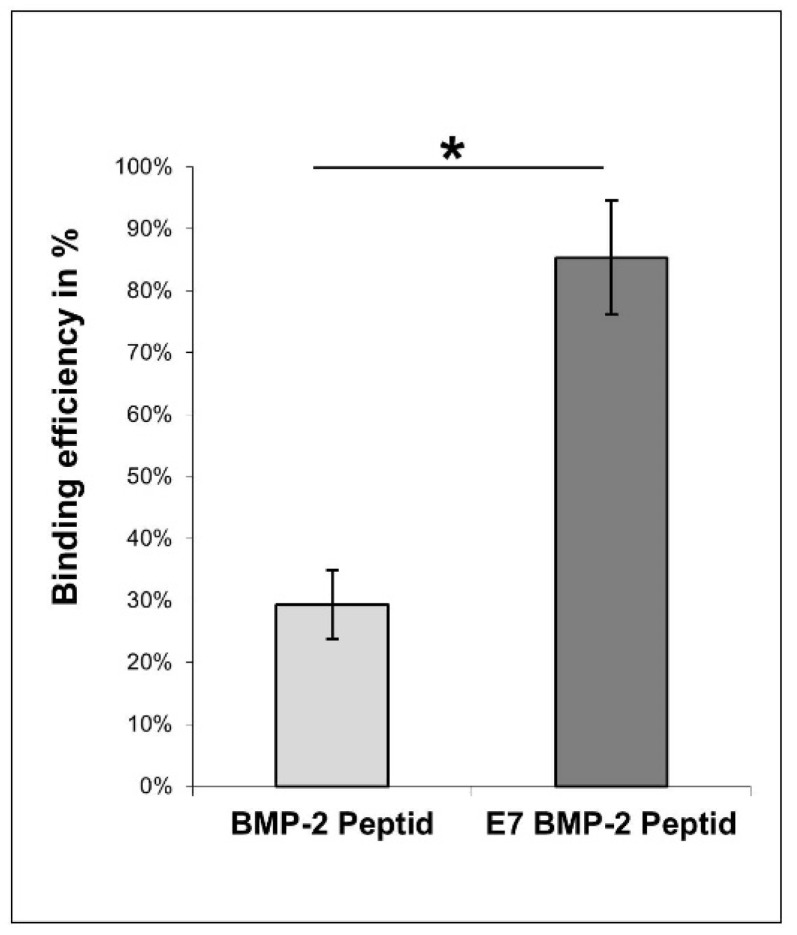
Indirect quantification of BMP-2 mimicry peptides binding to HA. BMP-2 mimicry peptides (1 mg/mL) with or without E7 Tag were incubated with equilibrated HA particles for 2 h at RT. Biochemical measurements of peptide concentration before and after incubation followed. Binding efficiency (in percent) compared to the initial applied peptide concentration is illustrated.* *p* < 0.001.

10 mg of the hydroxyapatite powder (Fluidinova, Moreira da Maia, Portugal) was washed in TBS buffer (0.15 M NaCl/50 mM Tris/HCl, pH 7.4) and centrifuged for 10 min at 5000 rpm. An overnight incubation in TBS buffer followed for equilibration. After a further centrifugation step, an incubation step with 200 µl BMP-2 mimicry peptide solution with or without E7 Tag (1 mg/mL) with continuous shaking for 2 h at 1200 rpm, followed at RT. Peptide concentration was determined before and after the peptide incubation step using the Micro BCA^TM^ protein assay kit (Pierce, Thermo Scientific, Waltham, MA, USA) following manufacturer’s instructions. Absorbance measurements were performed using the ELx800 plate reader (BioTek, Bad Friedrichshall, Germany) at a wavelength of 550 nm. As illustrated in Figure 3, binding efficiency was calculated in percent (n = 6) compared to the peptide concentration before incubation with the HA particles.

### 2.4. Detection of BMP-2 Peptide Binding to HA by Quartz Crystal Microbalance Analysis

**Figure 4:**
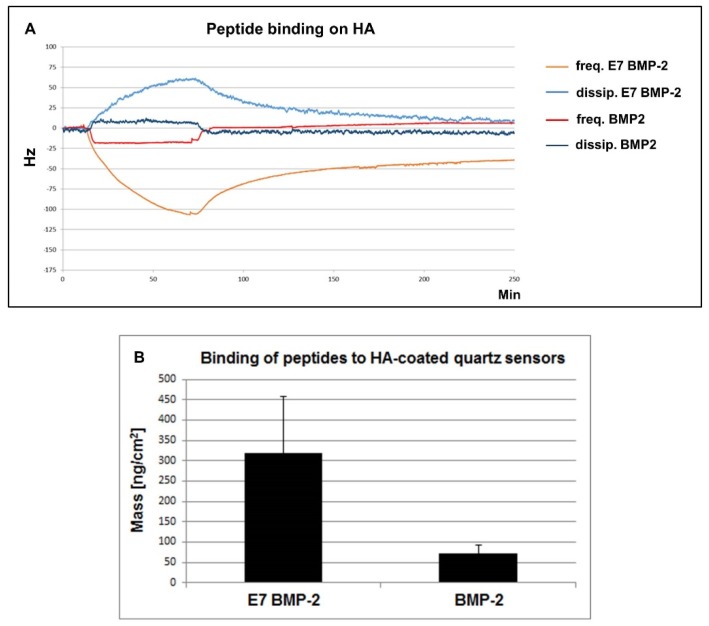
Direct detection of BMP-2 mimicry peptide binding on HA. BMP-2 mimicry peptides (0.6 mg/mL) flowed across the surface of the HA-coated quartz crystals for 45 min and washing with TBS buffer solution followed. (**A**) The red and orange curves illustrate the oscillation frequency and the light and dark blue curves illustrate the dissipation. Note the sudden increase (oscillation frequency) and decrease (dissipation) of the BMP-2 peptide curves after applying the washing buffer, indicating a nearly reversible adsorption-dissociation interaction of peptides without E7 Tag from the HA surface. In contrast, E7 BMP-2 peptides adsorb partly irreversible. (**B**) Quantification of the adsorbed mass of the BMP-2 peptides, with or without E7 Tag, to the HA-coated quartz sensors.

10 MHz quartz sensors with a 30 nm hydroxyapatite (HA) coating (3T Analytik, Tuttlingen, Germany) were mounted into the flow-cell of a qCell T quartz crystal microbalance (QCM) (3T Analytik, Tuttlingen, Germany). As previously described in detail [21], this acoustic sensing system with frequency and dissipation output allows real-time detection of mass- and damping sensitive (macro)molecular and even bacterial and cellular surface interactions. For all QCM runs, a flow rate of 110 µL/min was adjusted by means of a digital peristaltic pump (Reglo Digital MS-4/12, Ismatec, Wertheim, Germany). TBS buffer solution run across the quartz sensor surface for one hour to achieve equilibration of the signal base lines. Thereafter, the BMP-2 mimicry peptide solution (0.6 mg/mL in TBS), with or without Tag, flowed across the HA sensor surface for 45 min. To test the peptide binding strength, TBS buffer was applied for additional 3 h with the same flow rate. The recorded signal curves (Figure 4) represent the oscillation frequency (red and orange curve) and the dissipation (light and dark blue curve). Mass loading by peptide binding to the sensor surface resulted typically in a decrease of oscillation frequency and an increase in dissipation. During rinsing, unchanged signal levels indicate irreversible binding, whereas signal runs toward the original baseline level disclose partly or fully reversible peptides dissociation.

### 2.5. Alkaline Phosphatase (AP) Expression Analysis by Fluorescent Immunocytochemistry

**Figure 5:**
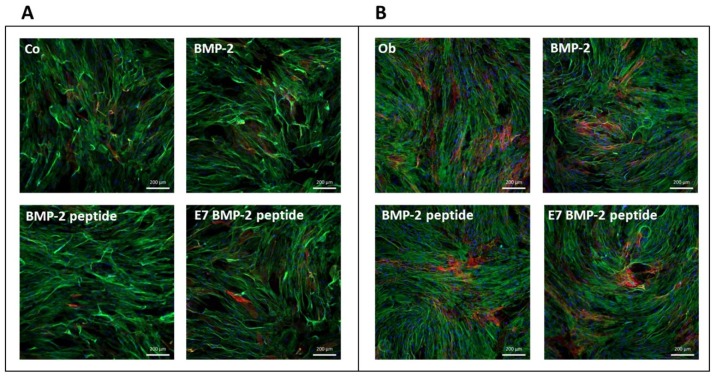
Representative pictures from three independent experiments for the detection of alkaline phosphatase (AP) expression by JPCs (red) with a DAPI (nuclei staining - blue) and phalloidin (cytoplasmic staining - green) staining. Cells were cultivated in 2D culture plates in the absence or presence of recombinant BMP-2 or BMP-2 mimicry peptides with or without E7-Tag under normal (**A**) or osteogenic conditions (**B**) and alkaline phosphatase activity (red) was detected.

5 × 10^3^ JPCs were seeded per well of the 96-well plates and cells were cultivated under normal (Co) and osteogenic conditions (Ob) with or without recombinant BMP-2 protein (100 ng/mL) or BMP-2 and E7 BMP-2 mimicry peptides (100 µg/mL) for 10 days. Cell monolayers were washed and fixed with 4% formalin for 15 min. at RT. After additional wash steps, cell monolayers were incubated for 1 h at RT with blocking buffer (PBS/1% BSA (bovine serum albumin)/5% normal goat serum provided by Jackson Immuno Research, West Grove, PA, USA). Thereafter, incubation with primary antibodies (monoclonal mouse anti-human alkaline phosphatase (AP), diluted 1:60 in PBS/1% BSA/1.5% normal goat serum, Bio-Techne, Minneapolis, MN, USA) for 2h at RT followed. After three wash steps with PBS, incubation with secondary antibodies (goat anti-mouse Cy3 labelled, Jackson Immuno Research) at a 1:300 dilution, followed for 1 h at RT. Cytoskeletal staining was then performed by incubation with AlexaFluor 488 phalloidin (Molecular Probes, Thermo Fisher Scientific, Waltham, MA, USA) for 20 min and DAPI (Sigma-Aldrich, Steinheim, Germany) for further 10 min. Representative images are illustrated in Figure 5.

### 2.6. Detection of Cell Mineralization in the Presence of Recombinant BMP-2 and BMP-2 Mimetic Peptides

**Figure 6:**
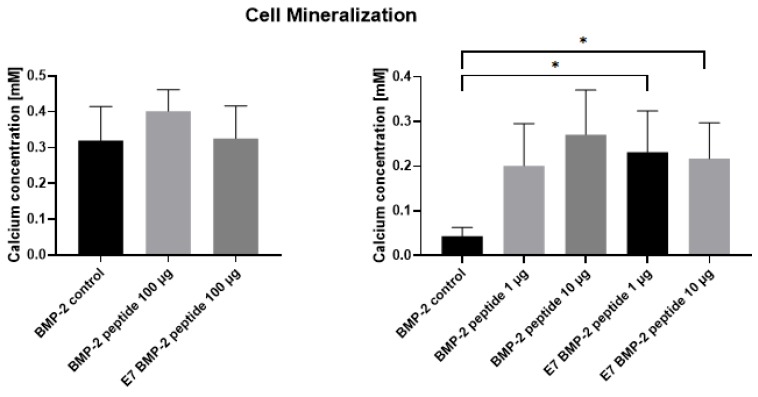
Quantification of JPC mineralization. JPCs were cultivated in the absence or presence of recombinant BMP-2 or BMP-2 mimicry peptides with or without E7-Tag (100 µg/mL) under osteogenic conditions (left panel). After Alizarin staining, calcium concentrations (mM) in JPC monolayers were quantified. By using BMP-2 mimicry peptides with or without E7-Tag in lower concentrations (1 and 10 µg/mL, right panel), significant higher JPC mineralization was detected in comparison to BMP-2 control (* *p* < 0.05).

JPCs were cultured for at least 20 days in the presence of recombinant BMP-2 protein (100 ng/mL) and/or with BMP-2 or E7 BMP-2 mimetic peptides (100 µg/mL). Cell mineralization was then detected by alizarin staining and quantification. Briefly, cultured JPC monolayers were fixed with zinc formaline for 15 min at RT. After washing with PBS, cells were stained with a 4 mM Alizarin solution for 30 min at RT. After intensive washing with aqua dest, cell monolayers were incubated with 10% acetic acid for 30 min while shaking, scraped from the plate bottom and cell suspensions were heated at 85 °C for 10 min. Samples were cooled down on ice for 10 min and centrifuged for 15 min at 15,000× *g*. Supernatants were used for quantification (Figure 6) after neutralization with 10% ammonium hydroxide using photometric measurements (ELx800, BioTek Instruments GmbH, Bad Friedrichshall, Germany) at a wavelength of 405 nm.

### 2.7. Detection of Metabolic Activities of JPCs Growing within Collagen/HA Composites

**Figure 7:**
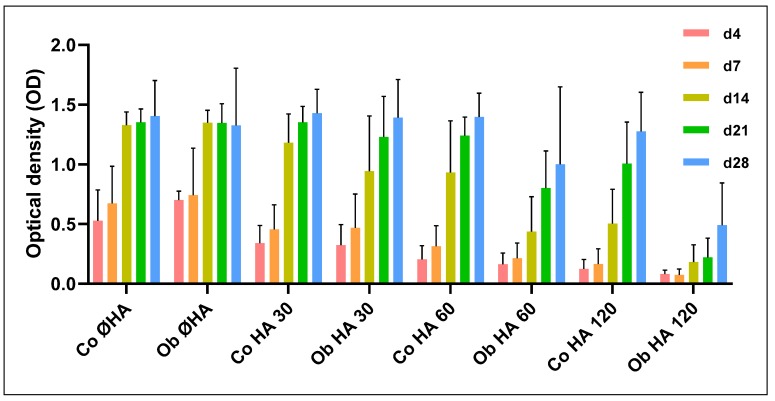
Detection of the optimal amount of HA for collagen/HA composites. Collagen gels or collagen/HA composites were prepared in a ratio of 1:1 (HA 30); 1:2 (HA 60), and 1:4 (HA 120). JPCs were cultured for 4–28 days within these constructs and metabolic activities were measured (at least 3 independent experiments). Optical densities (OD) of the measurements are illustrated. Note the decrease in cell viability with increasing HA amounts.

**Figure 8:**
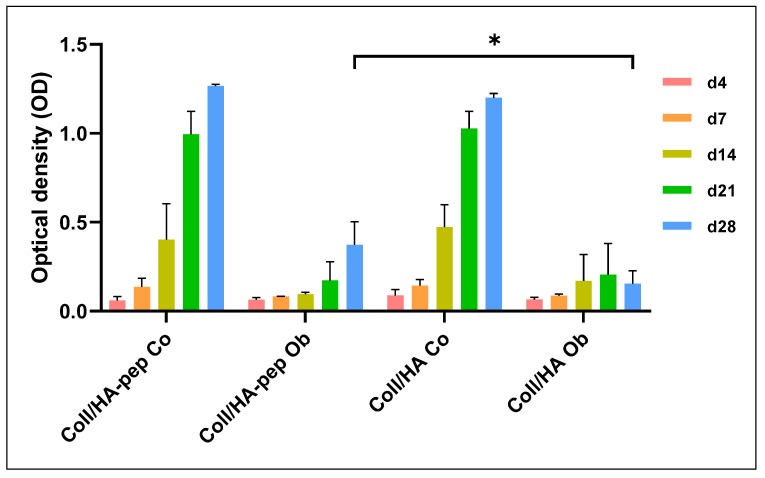
JPC proliferation within collagen/HA (1:2) composites. Collagen/HA composites in a ratio of 1:2 were prepared with (-pep) or without E7 BMP-2 peptides. JPCs were cultured within the composites under normal (Co) and osteogenic (Ob) conditions for 4–28 days (at least three independent experiments). Metabolic activities after these time points are illustrated. * *p* < 0.05.

JPCs were cultured for 4, 7, 14, 21, and 28 days within the collagen/HA composites consisting of different collagen/HA ratios (1:1–Coll HA 30; 1:2–Coll HA 60; 1:4–Coll HA 120) as shown in Figure 7 or for the same time periods within 1:2 collagen/HA composites with (30 mg collagen/60 mg HA/1200 µg peptides) or without E7 BMP-2 mimicry peptides, as shown in Figure 8.

At the above mentioned examination times, culture medium was replaced by 200 µL fresh medium and 20 µL of the substrate provided by the EZ4U cell viability kit (BIOMEDICA Medizinprodukte GmbH & KO KG, Vienna, Austria). After 4 h of incubation, optical densities were measured at 450 nm with a reference wavelength of 630 nm, using an ELx800 plate reader (BioTek, Bad Friedrichshall, Germany).

### 2.8. Live/Dead Staining of JPCs Growing within Collagen/HA (1:2) Composites

**Figure 9:**
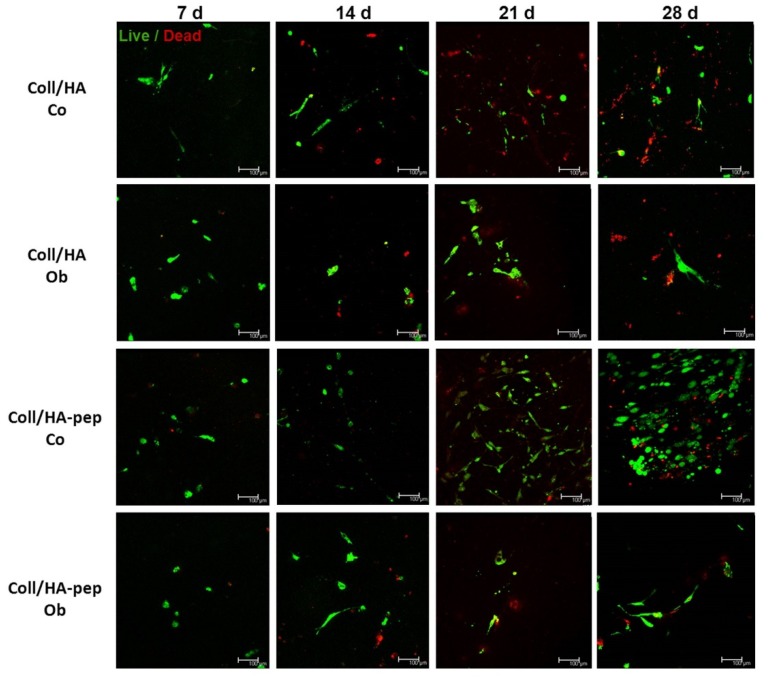
Live/dead staining of JPCs growing within collagen/HA (1:2) composites for different examination time points. JPC viability within collagen/HA (1:2) scaffolds was visualized by a fluorescent live/dead staining. Green fluorescence indicates intracellular esterase activity (living cells) and red fluorescence with ethidium-homodimer 1 indicates the loss in plasma membrane integrity (dead cells). The incorporation of E7 BMP-2 mimicry peptides promotes the long-term survival of seeded JPCs in particular under normal conditions (after 21 and 28 days of cultivation).

Live/dead labeling of JPCs growing within collagen/HA composites was performed using a staining kit containing membrane-permeant calcein (for cytoplasmic green fluorescent staining—cleavage of calcein by esterase activity in living cells) and membrane-impermeant homodimer-1 dye (red fluorescent staining of nucleic acid of membrane-compromised cells). Staining procedure was performed as recommended by the manufacturer (Invitrogen/Thermo Fisher Scientific, Waltham, MA, USA) and stained cells were visualized using confocal laser scanning microscopy TCS SP5 (Leica, city, Germany). Representative pictures are shown in Figure 9.

### 2.9. Statistic Analyses

For the data evaluation, means ± standard deviations are expressed. Statistical analysis was carried out using two-tailed student’s t-tests (detection of peptide binding on HA), or one-way ANOVA (cell mineralization), and two-way ANOVA (proliferation analysis) corrected for multiple comparison using Tukey’s tests. A *p*-values < 0.05 was considered significant.

## 3. Results

### 3.1. Detection of BMP-2 Mimicry Peptide Binding on HA

#### 3.1.1. Biochemical Quantification

Binding efficiency of BMP-2 and E7 BMP-2 mimicry peptides on HA was quantified indirectly after 2 h of incubation by colorimetric measurements of unbound peptides in the supernatants.

As illustrated in Figure 3, 29.00 ± 0.06% of the applied BMP-2 peptides were detected to bind to HA while almost the three-fold amount (85.00 ± 0.09%) of E7 BMP-2 mimicry peptides was shown to be bound to HA. The difference was highly significant (n = 6, *p* < 0.001).

#### 3.1.2. Quartz Crystal Microbalance

Binding efficiency of BMP-2 and E7 BMP-2 mimicry peptides was analyzed additionally by the mass sensitive quartz crystal microbalance (QCM) approach using hydroxyapatite-coated quartz crystals.

Representative curves from recorded QCM measurements are shown in Figure 4A. The figure illustrates E7 BMP-2 (light blue and orange curves) and BMP-2 (dark blue and red curves) mimicry peptide binding behavior to HA quartz crystals.

Oscillation frequency (red and orange curves) and dissipation (light and dark blue curves) reveal opposite curve progressions. Whereas oscillation frequency decreases, dissipation increases gradually during the first hour of continuous application of the peptide solution, indicating continuous E7 BMP-2 peptide binding to the HA surface of the sensor (light blue and orange curves). Afterwards, during crystal rinsing with TBS oscillation frequency increases while the dissipation decreases slightly in time and both curves achieve a nearly equilibrated state.

The dark blue and red curves reflect the interaction of the BMP-2 mimicry peptides to the HA surface of the quartz crystal. Initially, a slight oscillation frequency decrease and dissipation increase could be detected, reaching very quickly a steady-state during the whole peptide application time. Upon rinsing with TBS, the interaction showed complete reversibility. This result indicates almost immediate dissociation of BMP-2 peptides without E7 Tag from the HA surface.

Figure 4B represents the quantification of the resulting changes in mass (ng/sqcm) after the application of E7 BMP-2 (n = 8 measurements) and BMP-2 (n = 6 measurements) mimicry peptides to HA-coated quartz sensors. The significant higher mass resulting after application of E7 BMP-2 peptides (319 ± 138.70 ng/cm^2^) in comparison to the obtained mass after BMP-2 peptide application (73 ± 20.28 ng/cm^2^) to the quartz sensors, reflects an approximately 4.4-fold higher peptide binding of BMP-2 peptides containing the Tag, confirming the results obtained by the biochemical analysis.

### 3.2. Detection of AP Expression by JPCs Cultured in the Presence of BMP-2 Protein or Mimicry Peptides

We analyzed AP surface expression levels in untreated (A) and osteogenically stimulated (B) JPCs by semi-quantitative fluorescent immunocytochemical staining. As illustrated in Figure 5, JPCs showed similar AP levels and no relevant differences were detected in JPCs cultured in the presence of the entire BMP-2 protein or in the presence of BMP-2 peptides with or without E7 Tag.

### 3.3. Detection of JPC Mineralization in the Presence of BMP-2 Protein or Mimicry Peptides

As shown in Figure 6, detected calcium concentrations in JPC monolayers cultivated with unmodified and modified (E7 Tag) BMP-2 mimicry peptides were found to be in the tendency the same (BMP-2 control: 0.32 ± 0.1; BMP-2 peptide: 0.40 ± 0.06; E7 BMP-2 peptide: 0.33 ± 0.09 mM) compared with those detected in JPC monolayers stimulated with the whole BMP-2 protein (100 ng/mL), when peptides were used in high concentrations of 100 µg/mL (Figure 6, left panel). By using lower peptide concentrations of 1 or 10 µg/mL (Figure 6, right panel), JPC mineralization seemed to be significantly enhanced (* *p* < 0.05) compared to mineralization degree detected in the presence of the recombinant protein (BMP-2 control: 0.04 ± 0.02; BMP-2 peptide 1 µg/mL: 0.2 ± 0.09; BMP-2 peptide 10 µg/mL: 0.27 ± 0.1; E7 BMP-2 peptide 1 µg/mL: 0.23 ± 0.09; E7 BMP-2 peptide 1 µg/mL: 0.22 ± 0.08 mM).

### 3.4. Proliferation Analysis of JPCs Growing within Collagen/HA Scaffolds of Different Composition

To examine the optimal amount of HA for the preparation of the collagen/HA composites, JPCs were grown within the scaffolds containing different amounts of HA (collagen/HA ratios of 1:1 (HA 30); 1:2 (HA 60; 1:4 (HA 120)). Collagen scaffolds without HA served as controls (Co Ø HA/Ob Ø HA). All statistical significance tests were calculated compared to these controls.

As shown in Figure 7, cell viability decreases with increasing HA amounts both under normal and osteogenic culture conditions. JPCs showed similar proliferation activities within the collagen control without HA (Ø HA) and collagen/HA composites of a 1:1 (HA 30) ratio. A clear tendency of decreasing JPC proliferation rates were observed within composites of a 1:2 (HA 60) and 1:4 ratio (HA 120). In particular, osteogenically induced JPCs (Ob samples) seemed to be significantly diminished in their proliferation potential (*p*-value < 0.05 for all examination time points (with exception of day 4)), see Table 2 for the list of all calculated *p*-values).

### 3.5. Proliferation Analysis of JPCs Growing within Collagen/HA (1:2) Scaffolds

We decided to further prepare the collagen/HA composites of a ratio of 1:2 (similar to that found in the natural bone) for the incorporation of E7 BMP-2 peptides. As illustrated in Figure 8, osteogenic culture conditions of JPCs cultivated within the 1:2 collagen/HA composites showed strongly and significantly diminished proliferation activities compared to untreated (Co) constructs at all late time points (day 14, 21, and 28).

Comparing osteogenic culture conditions (Ob) with each other, significantly higher proliferation rates of JPCs within composites including E7 BMP-2 peptides (Coll/HA-pep) compared to those without E7 BMP-2 peptides could be observed after 28 days of culture.

This difference could be clearly visualized by CLSM images, as shown in Figure 9. The performed live/dead staining revealed higher amounts of living cells within Coll/HA composites containing E7 BMP-2 peptides, especially under normal culture conditions.

## 4. Discussion

In order to imitate physiological environment, intensive efforts have been undertaken to functionalize the biomaterial surfaces with components of the extracellular matrix. A novel trend is to use of synthetic functional peptides instead of whole proteins to keep costs low and to facilitate later approval issues. BMP-2 mimetic peptides seem to trigger osteogenic differentiation of human bone marrow MSCs, as shown after simple functionalization of glass slides [22]. In a recent study, Oki and co-workers succeeded in the development of thiol-maleimide clickable alginate microcapsules containing biomimetic RGD and BMP-2 peptides [23]. Encapsulated mouse pre-osteoblastic cells started osteogenic differentiation when BMP-2 mimetic peptides were conjugated to microcapsules without the addition of further osteogenic agents. Developing and transferring this usable approach into the human system and thereafter into clinical practice, small bone defects could be regenerated. However, the biggest challenge for oral and maxillofacial surgeons remains tissue regeneration of large bone defects as they occur, for example after tumor resections.

Inorganic compounds such as hydroxyapatite among other calcium phosphates are frequently used for the fabrication of bone substitutes for tissue engineering purposes either in combination with polymers [24,25,26,27,28,29] or with collagen to mimic the natural bone composition [30,31,32,33,34,35]. In a recent work of Linh and co-authors, the surface of porous hydroxyapatite scaffolds was modified by collagen treatment and BMP-2 conjugation [36]. Choosing this strategy, the use of crosslinkers is still needed and only a surface functionalization is obtained.

In the present study, we report on an approach for the generation of collagen/HA composites, simply biofunctionalized with BMP-2 mimicry peptides pervading the whole material. We tested the binding efficiencies of BMP-2 mimicry peptides to hydroxyapatite and detected biochemically three-fold higher binding levels of peptides with E7-Tag compared to those obtained by peptides without this poly-glutamic acid residue. Moreover, we tested the stability of the electrostatic binding by real-time QCM-analysis, based on measurements of small mass changes on the sensor surface in the nanogram range with a sensitivity for the applied system of 0.87 ng Hz^−1^ [21]. The QCM approach has meanwhile evolved from a simple acoustic based mass sensor to a powerful label-free bioanalytical tool [37]. QCM with dissipation detection (QCM-D) uses two independent quantifiable signals, the oscillation frequency and the energy dissipation. The frequency response is in our case related to the detected mass of surface bound peptides and the dissipation response is related to the viscoelastic properties of these adsorbed peptides, indicating rigidity (high dissipation) or softness (low dissipation) of these films. Using this approach, we detected fully reversible interaction of peptides without E7-Tag with the hydroxyapatite surface of quartz crystal sensors, whereas a more stable binding of E7-Tag peptides to HA surfaces in a partly reversible interaction could be proved. Assuming here proportionality of the frequency data and the adsorbed mass according to the Sauerbrey equation, a 4.4-fold higher binding of E7 BMP-2 to HA compared to the peptide without E7-Tag could be shown. The biological activity of the used BMP-2 peptides was tested and alkaline phosphatase induction was similar to that obtained by recombinant BMP-2. Quantification analysis performed with JPCs from three donors revealed slightly increased alkaline phosphatase activities compared to cells cultivated without peptides. Compared with JPCs cultured in the presence of BMP-2, two of them showed higher, one of them lower AP levels indicating a patient-dependent response (data not shown). This observation could be a limiting factor for the use of BMP-2/BMP-2 mimetic peptides based on the fact that not all patient cells are BMP-2 responders. A previous BMP-2 responding test should be carried out before clinical application, also in the sense of individualized medicine.

Unfortunately, we failed to detect cell mineralization within the Coll/HA/pep scaffolds based on high interference with HA particles. However, by alizarin quantification, we could clearly show that lower peptide concentrations have a higher impact in cell mineralization of osteogenically induced 2D cultures. Probably, peptides interfere or hinder each other spatially when concentrations are too high.

After preparation of collagen/HA composites of different ratios, we concluded that high HA amounts diminished JPC proliferation gradually. This finding confirms reports from other groups. In a recent study, the incorporation of nano-HA and micro-HA particles to polymer (polycaprolactone) scaffolds has been investigated [38]. The addition of nano-HA particles enhanced the adhesion, viability, and alkaline phosphatase activities of human mesenchymal stem cells at higher levels compared to the induction obtained by addition of micro-HA particles. In a study conducted by Cao and co-authors [39], increasing ratios of nano-HA within collagen/HA scaffolds on the surface of carbon/carbon composites had no significant impact on the proliferation activities of MC3T3 cells in vitro. However, Endo and co-workers detected decreased cell numbers with increasing amounts of octacalcium phosphate (OCP) [40] particles within the alginate/OCP microbeads.

The results obtained by our proliferation assays revealed significantly decreased cell viabilities in particular within 1:4 collagen/HA composites under osteogenic conditions in comparison to collagen matrices without HA. For this reason, we decided to further analyze JPC proliferation in more detail within 1:2 collagen/HA composites with or without E7-Tag BMP-2 mimicry peptides. Peptide biofunctionalization of 1:2 collagen/HA composites led to comparable JPC proliferation activities between 4 and 21 days in culture. However, significantly increased proliferation activities were detected after 28 days under osteogenic culture conditions (and the same tendency without reaching significance, under normal culture conditions) compared to JPCs growing within composites without peptides, as shown in Figure 8. Thus, E7-Tag peptides seemed to favor the long-term survival of JPCs. BMP-2 is a known protein promoting cell proliferation and differentiation however, heterogeneous osteogenic response of MSCs to recombinant human BMP-2 was reported [41], in line with our own previous experiences from JPCs. Padiolleau and co-authors demonstrated that mimetic peptides and geometrical cues direct MSCs fate [42]. For future studies, it would be conceivable to test the combination of several peptides that can synergistically regulate and/or accelerate osteogenic differentiation.

## 5. Conclusions

In our study, we present a successful approach for simple functionalization of biomaterial composites and demonstrate the suitability of the herein developed collagen/HA composites for the use as a vehicle to activate and deliver JPCs to sites where bone regeneration is needed. The incorporation of the used BMP-2 mimetic peptide seems to promote long-term survival of JPCs within the scaffold as well as cell mineralization in 2D cultures. In order to improve the efficacy of the composites and to mimic extracellular microenvironment more realistically, key osteogenic activators should be combined for future scaffold functionalization.

## Figures and Tables

**Table 1:** List of components for the preparation of collagen/HA composites. GNS = gel neutralization solution; HA = hydroxyapatite.

Collagen Solution 10 mg/mL	2x GNS	DMEM/20% FCS	HA	Peptide
3 mL	375 µL	375 µL	30 mg	600 µg
3 mL	375 µL	375 µL	60 mg	1200 µg
3 mL	375 µL	375 µL	120 mg	2400 µg

**Table 2:** Statistical ANOVA analysis of JPC proliferation activities calculated in comparison to cells growing within collagen scaffolds without HA (groups Co Ø HA and Ob Ø HA from Figure 7). n.s. = not significant; Co = untreated control; Ob = osteogenically induced samples.

	Day 4	Day 7	Day 14	Day 21	Day 28
Co HA 30 (1:1)	n.s.	n.s.	n.s.	n.s.	n.s.
Ob HA 30 (1:1)	n.s.	n.s.	n.s.	n.s.	n.s.
Co HA 60 (1:2)	n.s.	n.s.	n.s.	n.s.	n.s.
Ob HA 60 (1:2)	n.s.	n.s.	*p* < 0.001	n.s.	n.s.
Co HA 120 (1:4)	n.s.	n.s.	*p* < 0.01	n.s.	n.s.
Ob HA 120 (1:4)	n.s.	*p* < 0.05	*p* < 0.0001	*p* < 0.0001	*p* < 0.001
